# Impact of pharmacist-evaluated clinical decision support system alerts on potentially missing or inappropriately prescribed proton pump inhibitors at hospital discharge: a retrospective cross-sectional study

**DOI:** 10.1007/s11096-024-01746-6

**Published:** 2024-06-13

**Authors:** Lee Flückiger, Claudia Zaugg, Rico Fiumefreddo

**Affiliations:** 1 Hospital Pharmacy, Kantonsspital Aarau, 5000 Aarau, Switzerland; 2 Department of Internal Medicine, Kantonsspital Aarau, 5000 Aarau, Switzerland

**Keywords:** Clinical decision support systems, Electronic health records, Hospital, Medication safety, Proton pump inhibitors, Patient safety

## Abstract

**Background:**

Proton pump inhibitors (PPIs) are among the most prescribed drugs. A clinical decision support system (CDSS) could improve their rational use.

**Aim:**

The impact of an electronic algorithm (e-algorithm) implemented in a CDSS on potentially missing or inappropriately prescribed PPIs at hospital discharge, its specificity and sensitivity, and the outcome of the alerts issued were analysed.

**Method:**

An e-algorithm continuously monitored patients of a tertiary care hospital for missing or inappropriate PPIs. Following relevance assessment by a pharmacist, the alerts raised were either displayed in the patients’ electronic record or dismissed. After a three-month period, all adult patients’ records were retrospectively reviewed for missing or inappropriate PPIs at discharge. The results were compared with a corresponding period before CDSS introduction. Sensitivity, specificity and outcome of alerts were quantified.

**Results:**

In a 3-month period with 5018 patients, the CDSS created 158 alerts for missing PPIs and 464 alerts for inappropriate PPIs. PPI prescribing was proposed 81 times and PPI termination 122 times, with acceptance rates of 73% and 34%, respectively. A specificity of 99.4% and sensitivity of 92.0% for missing PPIs and a specificity of 97.1% and a sensitivity of 69.7% for inappropriate PPIs were calculated. The algorithm reduced incidents of missing PPIs by 63.4% (*p *< 0.001) and of inappropriate PPIs by 16.2% (*p* = 0.022).

**Conclusion:**

The algorithm identified patients without necessary gastroprotection or inappropriate PPIs with high specificity and acceptable sensitivity. It positively impacted the rational use of PPIs by reducing incidents of missing and inappropriate PPIs.

**Supplementary Information:**

The online version contains supplementary material available at 10.1007/s11096-024-01746-6.

## Impact statements

- An electronic algorithm implemented in the electronic health record systems can be a powerful tool to improve medication safety and rational use of drugs.
- The designed algorithm successfully identified both patients at risk for gastrointestinal bleeding without a PPI and potentially inappropriate PPIs in absence of drug-related risk factors.
- The evaluation showed the potential of a CDSS to promote the rational use of PPIs by reducing missing or inappropriate PPIs.
- Reluctance to discontinue medication, as opposed to initiating a new drug, diminishes the impact of a CDSS on potentially inappropriate PPIs.

## Introduction

Proton pump inhibitors (PPIs) are among the most prescribed drugs worldwide. A systematic review of data from 23 mainly Western countries (North America, Europe and Oceania) found that nearly one-quarter of adults use a PPI [1]. PPIs are used to treat peptic ulcers, gastroesophageal reflux disease (GERD), heartburn and other conditions related to gastric acid [2–5]. They protect against gastrointestinal bleeding in the presence of underlying risk factors such as older age, use of NSAID or other drugs interfering with the clotting system [6].

Observational studies have raised concerns about a variety of PPI-associated adverse drug events (ADE) ranging from dementia to fractures and infections [7–10]. Evidence of this association is lacking [11] but physicians and patients are aware of these ADEs [12]. Thus, PPIs are unjustifiably discontinued in patients at high risk of gastrointestinal bleeding [13]. On the other hand, a high proportion of prescribed PPIs are used without a documented indication [14, 15].

This problematic PPI use is recognised: the Choosing Wisely initiative provides recommendations for patients with heartburn and GERD [16], national guidelines, such as those from the National Institute of Health and Care Excellence, the Beers List and in-house recommendations have attempted to improve the rational use of PPIs [17, 18]. However, there has been little or no change in prescribing behaviour [19].

Clinical decision support systems (CDSS) provide physicians with knowledge and patient-specific information to improve medication safety [20]. However, their performance in preventing clinically relevant medication errors is influenced by several factors, such as a high number of irrelevant alerts. It could be improved by better specified algorithms and alert criteria, as well as extended data delivery [19–21].

Several studies analysed improving under- and overprescribing PPIs with electronic tools. A Danish study found that a CDSS changed general practitioners’ (GPs’) prescribing patterns, resulting in an increase in co-prescribing of NSAIDs and PPIs for moderate or high risk of gastrointestinal bleeding (OR 1.33 and 1.58, respectively) [22]. There was also a small increase in guideline-concordant care due to CDSS in a similar American study that looked at missing PPIs in outpatient NSAID prescriptions [23]. At Leiden University Hospital, an algorithm was implemented to detect missing PPI medication needs in patients on NSAIDs over 70 years of age or in patients over 60 years of age with an additional risk factor for peptic ulcer. This system generated relatively few alerts and a detailed algorithm analysis was not performed [24]. A study in Germany aimed to reduce the long-term use of PPIs in a shared decision-making process between GPs and their patients using a CDSS [25]. To date, only qualitative results of this study have been published on the patients’ perspective on the used CDSS, but none on effectiveness [26]. To our knowledge, there is no published study of a CDSS aimed at improving under- and overprescribing of PPIs in an inpatient setting. However, as the studies above show, CDSS in the electronic health record (EHR) offers the potential to achieve this.

At the Kantonsspital Aarau, we use a CDSS that analyses patient data using electronic algorithms (e-algorithms). We have developed an e-algorithm to identify patients with drug-related risk factors for gastrointestinal bleeding but are not receiving a PPI, as well as patients with a potentially inappropriate PPI prescription.

### Aim

This study investigated the impact of an e-algorithm based CDSS implemented in the EHR on potentially missing or inappropriately prescribed PPIs at hospital discharge. The e-algorithm’s specificity and sensitivity and the outcome of alerts issued were analysed to assess its performance.

### Ethics approval

The study was conducted according to the guidelines of the Declaration of Helsinki and approved by the north-western and central Switzerland ethics committee on 14th July 2021 (Project-ID: 2021-01379).

## Method

### Setting

This study was conducted in a 669-bed tertiary care hospital in Switzerland. The hospital uses an in-house CDSS (“KPharm”) developed by a multidisciplinary team of physicians and pharmacists and directly implemented into the EHR (KISIM™ by CISTEC). KPharm is based on different e-algorithms that all allow for multiple alerts, reviewing all patient files hourly. Alerts can be displayed directly in the patient's record or evaluated first by a clinical pharmacist. A comprehensive explanation of this CDSS can be found elsewhere [27].

The PPI-e-algorithm identifies adult patients with a missing PPI prescription despite drug-related risk factors for gastrointestinal bleeding and patients with a PPI prescription in the absence of any drug-related risk factors. The e-algorithm focused on drug-related risk factors, as diagnoses were not documented in the EHR in a standardised format (e.g., ICD codes) during hospitalisation. The e-algorithm was developed based on current guidelines [2, 4, 5, 28]. Using prescription data from 2019, the e-algorithm was simulated and adapted to achieve a balance between clinical relevance and a manageable number of alerts (< 20 per day). Therefore, it considered intravenous PPI prescriptions and twice-daily prescriptions as appropriate. The e-algorithm comprised 11 different alerts for missing PPIs using several trigger conditions. They were ranked for clinical relevance; lower numbered alerts suppressed higher numbered alerts to avoid duplicate alerts. For inappropriate PPIs only one alert was specified, triggered by a PPI prescription in absence of any risk factors (see Table 1 for details). The e-algorithm was tested in an EHR test environment before deployment. The testing covered both positive and negative scenarios using defined use cases. The e-algorithm has been in use since July 1st, 2021.

**Table 1:** Alerts of the PPI e-algorithm

Alert no	Trigger conditions	
Alerts for missing PPIs		
1	NSAID and	Antiplatelet therapy
2	NSAID and	Therapeutic anticoagulation
3	NSAID and	Corticosteroid ≥ 10 mg prednisone equivalent
4	NSAID and	Drugs associated with gastrointestinal bleeding^a^
5	NSAID and	Age ≥ 65 years
6	NSAID and	Thrombocytes < 30 G/L
7	Therapeutic anticoagulation + dual antiplatelet therapy (DAPT)	And risk factor^b^
8	Therapeutic anticoagulation + low dose aspirin	And risk factor^b^
9	Dual antiplatelet therapy (DAPT)	
10	Low dose aspirin + corticosteroid ≥ 10 mg prednisone equivalent	And age ≥ 65 years
11	≥ 4 Risk factors^b^ present	
Alert for inappropriate PPIs		
12	PPI without any risk factors^b^ (excluding risk factor age ≥ 65 years)	

^a^Selective serotonin reuptake inhibitors (SSRIs) (list may be expanded in future)

^b^NSAIDs, COX2-inhibitors, antiplatelet therapy, therapeutic anticoagulation, drugs associated with gastrointestinal bleeding^a^, age ≥ 65 years, thrombocytes < 30 G/L

All alerts were first sent to a dedicated mailbox within the EHR and assessed by the clinical pharmacist on duty. The assessment during the study period was conducted by different pharmacists who had been provided an update on current guidelines for PPI usage for alert assessment. If clinically relevant, the alert was released for display in the patient’s record as a non-interruptive message, addressed to the ward physician. It contained information about the alert, trigger factors and a patient-tailored recommendation. If the conditions that triggered an alert were no longer valid, it was automatically terminated by the CDSS.

### Retrospective analysis of impact on missing or inappropriate PPI prescription

We conducted a retrospective observational study for a three-month period with the use of the e-algorithm (01.07.2021–30.09.2021) and a corresponding period without the use of it (01.07.2020–30.09.2020). All patient data (age, sex, length of stay, laboratory values) and prescription data (medication, administration details, chronology) were exported from the EHR. Patients aged < 18 years (as excluded in the CDSS) or who refused to give general consent for the use of their data generated during treatment were excluded from the impact analysis.

Patient records were screened for missing PPIs (in presence of drug-related risk factors for gastrointestinal bleeding) and inappropriately prescribed PPI (without an obvious indication in the patient record) on the day of discharge. The frequency of incidents for the period with the CDSS was compared to the period without. Characteristics were collected for patients discharged in both study periods to allow for comparison. The chi-square test was used to determine statistical significance. A *p*-value of < 0.05 was considered statistically significant.

Jupyter Notebooks (version 6.1.5), pandas (version 1.3.5) and ScyPy (version 1.7.1) for Python (version 3.9.2) were used for data aggregation, processing, and analysis. Patient records that were identified as potentially missing a PPI or having an inappropriate PPI at discharge underwent manual review to verify the need for PPI or documented indication.

### Analysis of acceptance rate of alerts, sensitivity, and specificity

All alerts issued by the CDSS during the observation period in 2021 were flagged as either *not relevant* (no intervention required or paused for later evaluation), *intervention* (message sent to clinician) or *problem resolved* (automatically terminated by CDSS as trigger conditions are no longer met). All interventions that resulted from PPI alerts were followed up. The intervention was defined as accepted if the responsible clinician at least partially complied with the suggested recommendation. Unaccepted or not-assessable interventions were labelled as such. The acceptance rate was calculated by dividing the number of accepted interventions by the number of all intervention messages sent.

The KPharm alerts were compared with the cases of missing or inappropriate PPI identified in the retrospective analyses to identify patients for which no alert was triggered. All cases were then assessed by the decision pathway as true/false and positive/negative and classified accordingly. Sensitivity and specificity were calculated by category (missing or inappropriate PPIs) and not for individual alerts.

## Results

During the period with the e-algorithm (01.07.2021–30.09.2021), a total of 5018 adult patients with 5534 hospitalisations were counted, of which 421 patients (8%) and 462 hospitalisations (8%) were excluded from the impact analysis because those patients refused consent. In the comparison period (01.07.2020–30.09.2020), 5044 patients with 5643 hospitalisations were counted, of which 416 patients (8%) and 465 hospitalisations (8%) were excluded for refusing consent.

Table 2 shows the characteristics of patients discharged during observation period with the e-algorithm and the reference period before its implementation. In both periods, about one third of the patients had a PPI prescription. There was no significant difference in selected characteristics between the two study groups.

**Table 2:** Characteristics of patients of both observation periods

Observation period	2020 Without e-algorithm	2021 With e-algorithm	*P-*value^a^
	01.07–30.09	01.07–30.09	
Number of patients	4628	4597	
Male	2258 (48.8%)	2259 (49.1%)	0.752
Female	2370 (51.2%)	2338 (50.9%)	
Per age group			
18–30	468	495	0.752
30–55	1373	1362	
55–65	749	747	
≥ 65	2038	1993	
Mean age in years	58.7 ± 20.1	58.3 ± 20.2	
Inpatient cases	5178	5072	
Cases with PPI prescriptions	1670 (32.3%)	1674 (33.0%)	0.428
Duration of stay in days			
0–3 days	1737	1825	0.204
3–6 days	1632	1564	
6–15 days	985	942	
> 15 days	274	266	
Mean duration of stay in days	5.6 ± 6.3	5.4 ± 6.0	
Number of concomitant drugs			
0–5	1453	1509	0.117
6–10	1735	1731	
10–15	1012	957	
16–20	538	489	
> 20	440	386	
Mean number of concomitant drugs	10.3 ± 7.5	9.8 ± 7.0	
Number of cases with drug-related or other risk factors (based on trigger conditions)			
NSAIDs	442	443	0.758
COX2-inhibitors	10	9	0.964
Low dose aspirin	843	833	0.866
Antiplatelet therapy	188	185	0.994
Therapeutic anticoagulation	413	421	0.572
Corticosteroid ≥ 10 mg prednisone equivalent	49	45	0.834
SSRIs	199	222	0.189
Age ≥ 65 years	2343	2240	0.278
Thrombocytes < 30 G/L	29	39	0.238

^a^Chi-squared-test

Case numbers for drug-related or other risk factors are based on the same trigger conditions as used in the e-algorithm

A total of 622 alerts were generated during the observation period in 2021. Of these alerts, 25% (n = 158) concerned missing PPIs (alerts 1–11) and 75% (n = 464) concerned inappropriate PPI prescriptions (alert 12). Thus, in 3% of 5072 hospitalisations during the observation period, drug-related risk factors for gastrointestinal bleeding were detected without a PPI being prescribed.

**Fig. 1:**
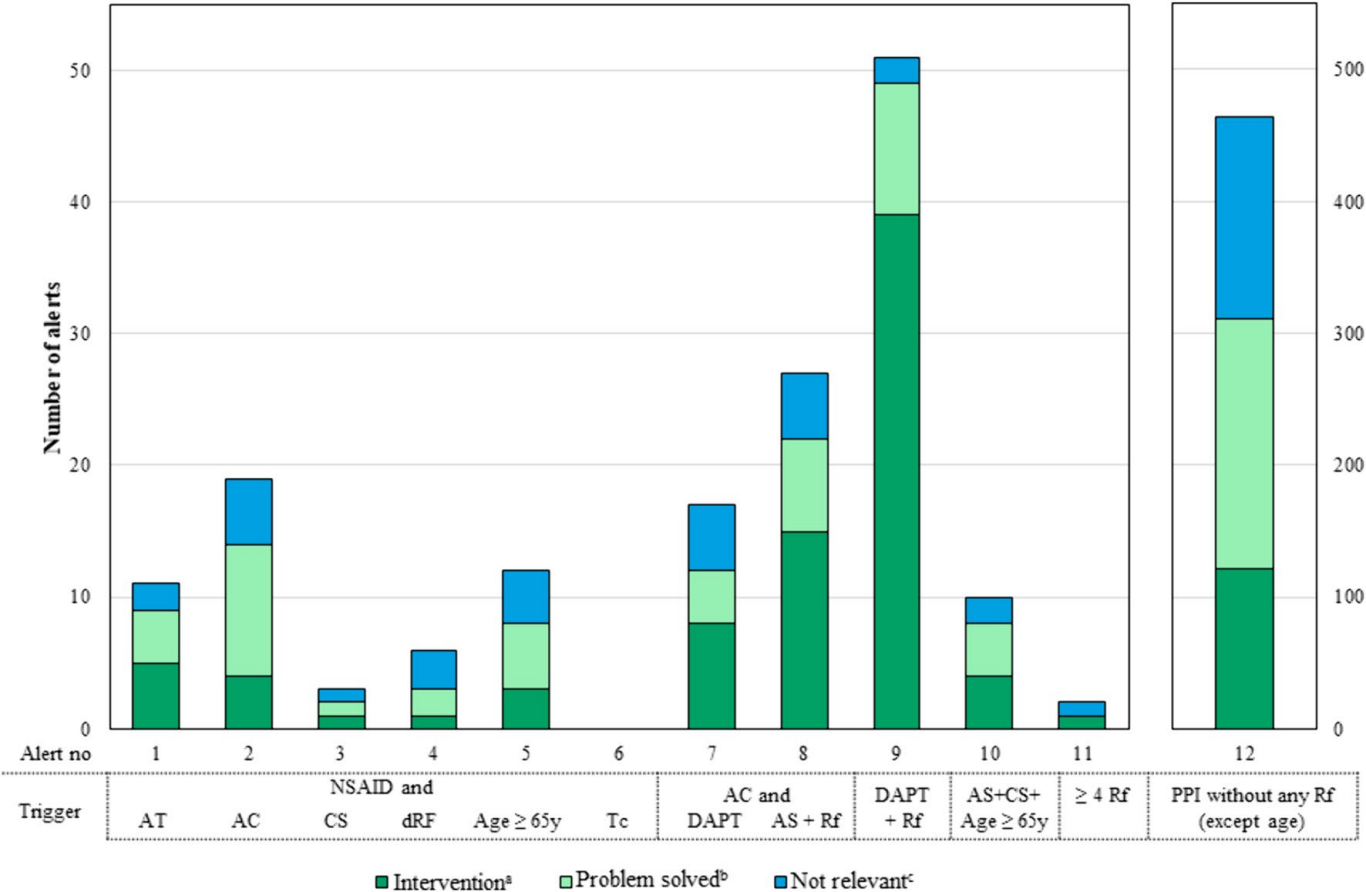
Number of triggered alerts per type (alert no) and assessment. **a** a message was sent to the physician with a recommendation. **b** Alerts automatically terminated by the CDSS were labelled as problem resolved. **c** Alerts labelled as not relevant were terminated by the clinical pharmacist. AT = antiplatelet drug, AC = anticoagulation in therapeutic dose, CS = corticosteroid ≥ 10 mg prednisone equivalent, dRF = drug associated with gastrointestinal bleeding, Tc = thrombocytes < 30 G/L, DAPT = dual antiplatelet therapy, AS = low dose aspirin, Rf = risk factor for gastrointestinal bleeding (drugs, age ≥ 65 years or Tc)

An alert for a potentially inappropriate PPI was raised in 9% of all hospitalisations and 27% of 1674 patients prescribed a PPI during their hospital stay (see Fig. 1).

68% of the alerts for missing PPIs were generated for a risk situation in the presence of antiplatelet therapy (alerts 7 to 11). The most common alert was for the combination of dual antiplatelet therapy (DAPT) and another risk factor (e.g., age ≥ 65 years). The remaining alerts related to NSAID use and another risk factor (alerts 1–6).

A total of 203 intervention messages (33% of all alerts) were sent to the physicians. Intervention messages were more frequently for missing PPIs (51%) than for inappropriate PPIs (26%). The proportion of alerts terminated was similar for both alert categories. More alerts for inappropriate PPIs were classified as not relevant (33% vs. 19%). Excluding automatically terminated alerts, 44% of alerts for inappropriate PPIs resulted in an intervention, compared to 73% of alerts for missing PPIs.

**Fig. 2:**
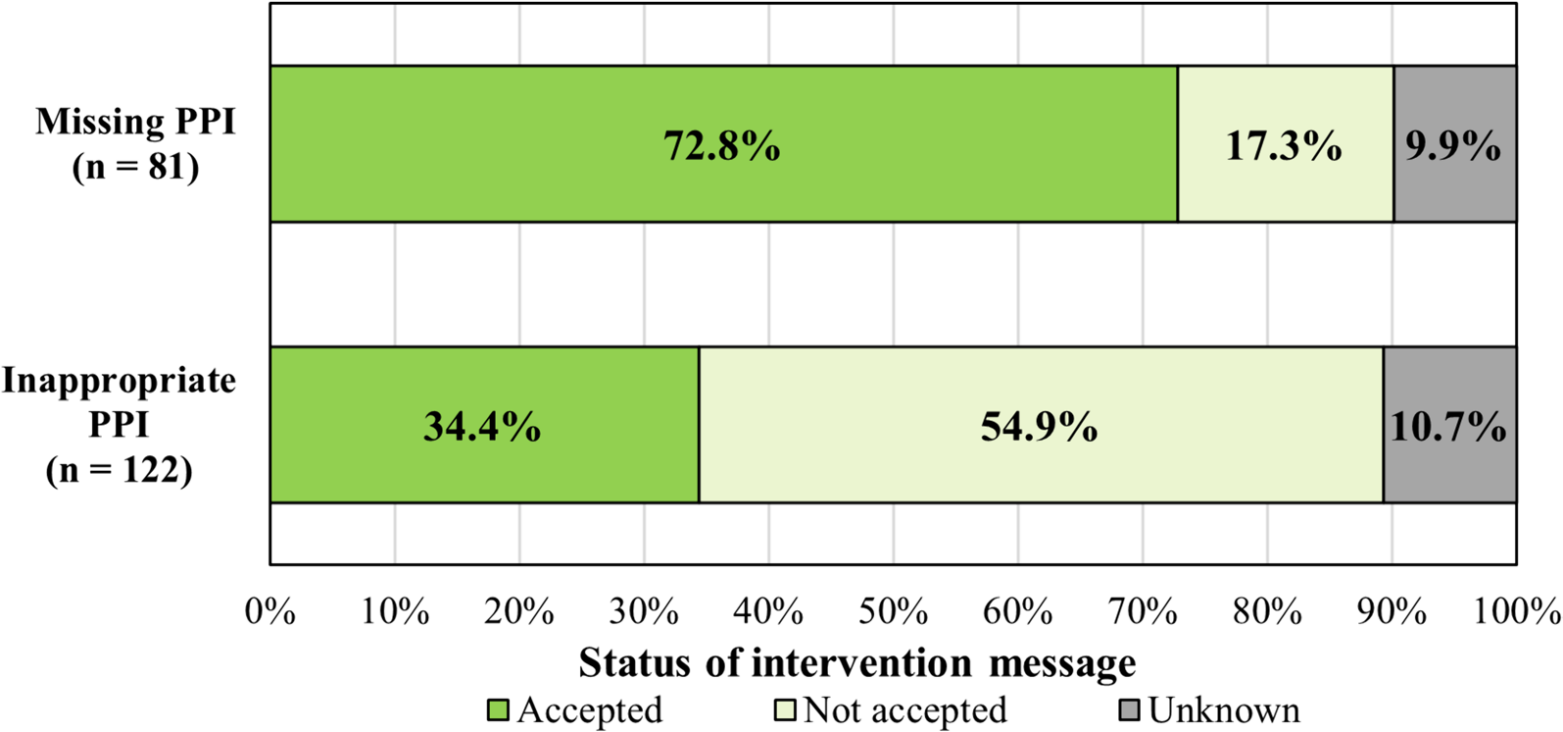
Acceptance rate of interventions messages. The acceptance is shown in percentages as accepted, not accepted or unknown (no follow up or not assessable) for missing PPIs (alerts 1–11) and inappropriate PPI (alert 12)

Overall, 50% of the recommendations were implemented by the physician, while for 10% of interventions, it was unclear whether the physician had considered the recommendation for the patient's treatment. However, there was a large difference between the two categories. Interventions for missing PPIs were accepted more frequently (73% of all alerts) than those for inappropriate PPIs (34% of all alerts) while the proportion of non-assessable interventions remained approximately the same (see Fig. 2).

**Fig. 3:**
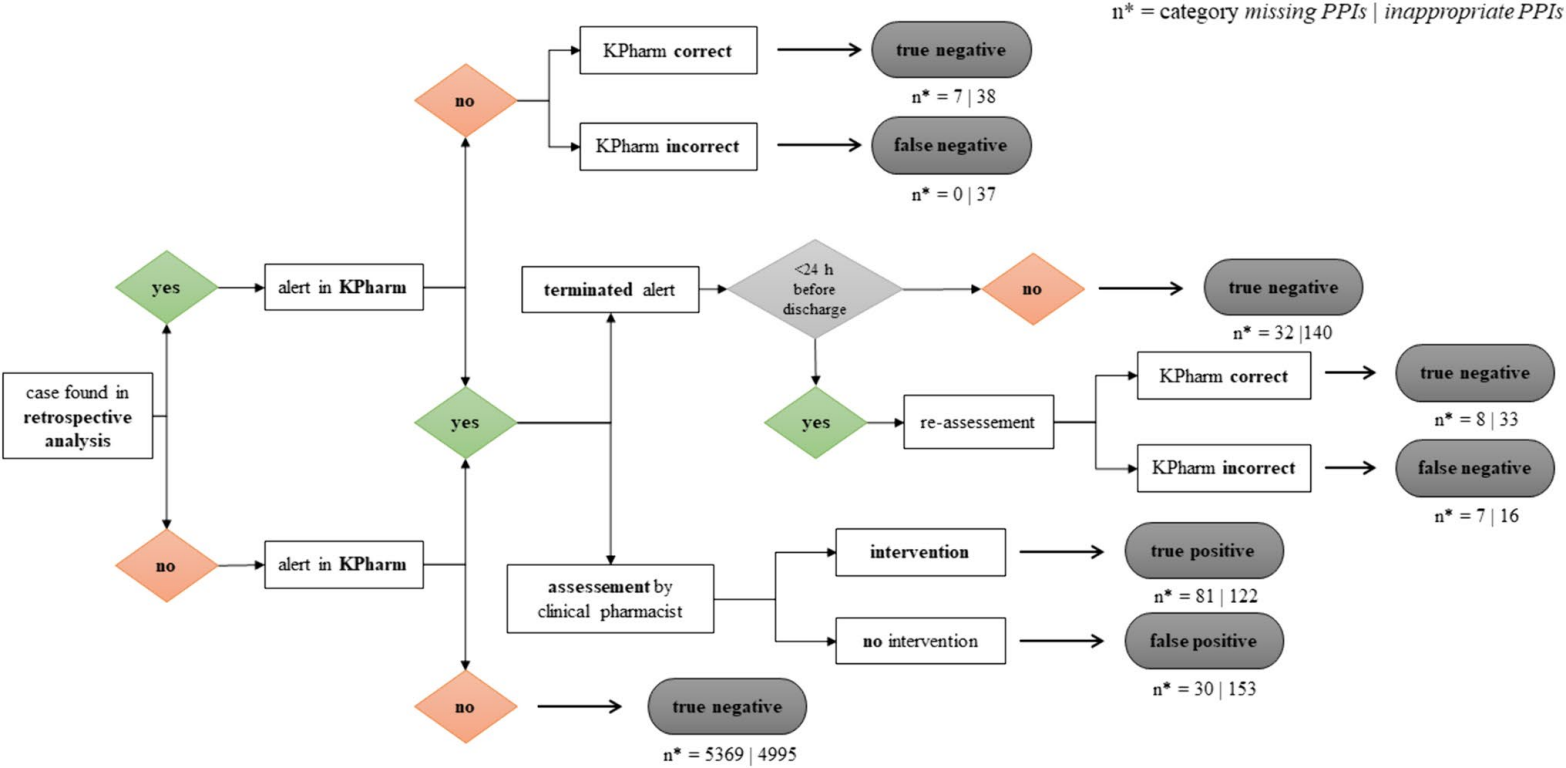
Sensitivity and specificity decision pathway and evaluation of cases

Specificity and sensitivity were calculated as shown in the decision pathway by category (see Fig. 3). The e-algorithm sensitivity and specificity for missing PPIs were 92.0% and 99.4% respectively. For inappropriate PPIs, specificity was 97.1% and sensitivity was 69.7%.

There was a highly significant reduction from 67 to 24 events (63%) in the missing PPIs category and a significant reduction from 329 to 270 events (16%) in the inappropriate PPIs category (see Table 3).

**Table 3:** Incidence of missing or inappropriate PPIs at discharge during the observation period without (2020) and with (2021) the e-algorithm

Category	Observation period 01.07.–30.09		*P-*value^a^	Relative reduction
	2020 Without e-algorithm	2021 With e-algorithm		
	Incidences			
Missing PPIs	67	24	< 0.001	− 63.4%
Inappropriate PPIs	329	270	0.022	− 16.2%
Cases without an incidence	4782	4778	< 0.001	–
Total cases	5178	5072	–	–

^a^Chi-squared-test

## Discussion

Our e-algorithm showed a similar specificity for missing and inappropriate PPIs (99.4% vs. 97.1%), while the sensitivity differed (92.0% vs. 69.7%). The lower sensitivity for inappropriate PPI resulted from a programming error producing false negatives, all related to the prescription of NSAIDs on demand. In developing the e-algorithm, it was decided that an on-demand NSAID should only be considered if taken more than six times in 48 h. Although on-demand prescriptions were included during testing, the programming error was not identified. In fact, another aim of the retrospective analysis was the identification of any programming weaknesses resulting in false negatives. This error has since been corrected. Other studies analysing the impact of a CDSS on rational use of PPIs did not report sensitivity and specificity, but in a scoping review, the clinical validation of several CDSS for drug-related problems showed a sensitivity ranging from 28 to 85% and a specificity from 42 to 75% [29]. Our results are in the upper range and align with the recommendation for another drug-related problem, that a CDSS should maximise specificity while keeping sensitivity at a value above 75% [30]. To avoid alert-fatigue, a high specificity is one of the main objectives of our CDSS [27].

The e-algorithm triggered 622 alerts during the three-month study period. Alerts for an inappropriate PPI were most frequent (75% of total alerts) but less relevant (intervention for 26% of alerts). The expected higher proportion of irrelevant alerts for inappropriate PPIs can be explained by the lack of coded diagnoses, a common issue in many Swiss hospitals. Natural language processing would be an alternative option we could not implement so far.

In our study we identified 7% of PPI prescriptions (122 interventions for 1674 prescriptions) as inappropriate. The literature suggests rates between 6.4 and 86.0% [15, 31, 32]. Our study probably underestimates the proportion of inappropriate PPIs due to the measures taken to reduce the alert burden generated by this e-algorithm. This was decided as a high alert volume could hinder the timely processing of more critical CDSS alerts, such as those related to anticoagulation, or contribute to alert fatigue for both pharmacists and physicians.

Our e-algorithm showed an overall intervention rate of 33%. Other CDSS with pharmacist involvement showed rates of 2.3% [33] or 20.1% [34]. One study analysing a CDSS screening for missing PPIs also reported intervention rates [24], but the low frequency of alerts in that study (3 in 6 months) precludes comparison.

The recommended initiation of a PPI was more accepted (73%) than the termination of a PPI prescription (35%), despite efforts to transmit only relevant alerts. The urology department for example prescribed PPIs as standard post-operative measure for stress ulcer prevention, despite reminders of the weak evidence for this practice [35, 36]. Physicians often described the PPI use as “lifestyle-use”, that the patient rejected the discontinuation. This is difficult to address by the e-algorithm. There is no comparable study for CDSS-based interventions for PPIs reporting acceptance rate; Yailian et al. [37] showed in a retrospective analysis of documented pharmacists’ interventions related to PPIs that “addition of a new drug” was more accepted (83.8%) than “drug discontinuation” (73.1%). While methodology differed heavily, they reported a higher acceptance rate than we achieved with our approach.

The impact of the e-algorithm on PPI use was assessed at discharge to establish a clear timepoint for comparison. While none of the selected characteristics differed significantly between the two periods studied, the quantitation of the e-algorithm’s impact was possible. The e-algorithm reduced the incidence of missing PPIs by 63%. The reduction was lower for NSAID alerts than for alerts regarding dual or single antiplatelet therapy with risk factors (alerts 7–9). Our results are consistent with the study by Gill et al. [23] reporting a significant increase in guideline-concordant treatment in high-risk patients on low-dose aspirin. However, they contrast the findings of Petersen et al. [22] reporting a CDSS effect on the co-prescription of PPIs and NSAIDs in medium- and high-risk groups, but not on the prescription of aspirin in the presence of risk factors.

The reduction of the incidence of inappropriate PPIs was low due to low acceptance rate, but still significant. Thus, the PPI e-algorithm had a positive impact on the rational use of PPIs in our hospital while using relatively low resource (a few minutes per alert) for a continuous monitoring of all adult hospital patients.

In summary, our findings match the results of intervention studies that have improved the rational use of PPIs. Coté et al. increased the use of gastroprotection in patients at risk from 43 to 61% [38]. Chen et al. improved the rate of rational use from 36 to 68% [32]. Nonetheless, the impact could further be improved by involving patients and physicians in the deprescribing process [32, 38, 39].

### Limitations

This study has several limitations. Alerts were assessed by different pharmacists. Although instructions for alert assessment were provided, outcomes may have varied depending on experience and workload of the responsible pharmacist, particularly for the relatively lower-priority PPI e-algorithm. Despite the short observation period of 3 months, over 4000 patients were monitored, which may mitigate this limitation. E-algorithm design was limited due to the unavailability of coded relevant diagnoses and other information to assess appropriateness. The e-algorithm also did not check for correct PPI dosage, nor did it distinguish between prophylactic and therapeutic uses. Detailed parameters, such as how long a prescription had to be valid to be considered as a risk factor or cut-off parameters, were not based on strict guidelines but on an interdisciplinary consensus in our hospital. Moreover, the impact of the e-algorithm was only assessed in terms of presence / absence of a PPI at discharge and not in terms of ADE. Finally, these findings derive from a single hospital thus, limiting the significance of its results.

## Conclusion

High specificity was achieved for both missing and inappropriate PPI alerts. Despite efforts to transmit only relevant alerts, the acceptance to deprescribe was low, achieving only a small reduction of inappropriate PPI at discharge. The reluctance to deprescribe cannot be addressed by CDSS alone but requires broader action. A significantly decreased incidence of missing PPIs was achieved. The e-algorithm could be extended to cover other issues related to PPI use. However, this must be seen in the overall context of the CDSS. The PPI algorithm is one of many and generates a high number of alerts, despite efforts to limit it. High alert volume could lead to more critical alerts not being processed timely or alert fatigue.

The PPI e-algorithm achieved our objectives and will continue to be used. Future studies could assess possible habituation to the interventions or teaching effects.

## Supplementary Information

Below is the link to the electronic supplementary material.

Supplementary file1 (DOCX 20 KB)
