# Supplementary material for: Impact of pharmacist-evaluated clinical decision support system alerts on potentially missing or inappropriately prescribed proton pump inhibitors at hospital discharge: a retrospective cross-sectional study
Source: Int J Clin Pharm. 2024 Jun 13;46(5):1143–51. doi: 10.1007/s11096-024-01746-6 (PMC11399224; doi:10.1007/s11096-024-01746-6)
Supplement: Supplementary file 1 — Supplementary file1 (DOCX 20 KB) [file 11096_2024_1746_MOESM1_ESM.docx]

**Simplified information on the e-algorithm**

Python source code and more elaborate explanation of developing this e-algorithm, uses cases for testing and pseudo-programming code can be found (in German) here: <https://www.gsasa.ch/deliver.cfm?f=0CD89DA59212A7CBAEDB92D04857B4B98B3073B687A929A4995EA5F1B9F08F77318BAE408BB7B6EB7B909BECEF6FA5A4B2FEC3C0BE9588AF929D5AB8BB448789404ABC4DC6CCECF08B178EA3A0039E54FF5AB3AAB48D5348599D959EF05E0BFC0C8D1B52424347F888&type=.pdf>

**The PPI algorithm detects the following problems:**

***Lack of PPI prescription with the following risk medications in combination or in the presence of other risk factors:***

- NSAIDs

- COX-2 inhibitors

- Platelet aggregation inhibitors (TAH)

- Anticoagulants in therapeutic dosing

- Glucocorticoids

- SSRIs and other medications associated with a risk of bleeding

(Age ≥ 65 years and thrombocytopenia are sought as additional risk factors)

***Proton pump inhibitors without indication***

**The following aspects are not checked in this algorithm:**

- Lack of PPI solely based on a diagnosis or pathogenesis

- Incorrect therapy regime for Heliobacter pylori eradication

- Interactions due to PPI (-> detected partially by other e-algorithms)

- Duplications of PPI (-> detected by e-algorithm for combination problems)

- PPI Dosage

**Considered patients:**

- Age >= 18 years
- Open inpatient case

**Prescriptions must fulfill the following conditions to be considered by the e-algorithm**

- On-demand prescriptions are not considered (except alert n2)
- Prescriptions with "?" as the quantity are evaluated as paused prescriptions.
- If there are several prescriptions that fulfil the condition for an ATC code or occur in one of the
- tables, only 1 prescription must fulfil all conditions.

**For alerts 1-11:**

The 2 drugs triggering the alert must be prescribed concomitantly for at least the last 24 hours for alerts 1,2, 7 and for alert 3 - 6, 8 – 11 for at least 48 hours

**For alerts 12:**

PPI: number of dosis per day < 2, route of administration NOT i.v.

No alert 12 in the last 365 days

**Considered PPI Prescription**

ATC Codes: A02BC*, A02BA*, A02BD*, M01AB55, M01AE52, B01AC67

**Considered NSAID Prescription**

ATC Code N02BA01, M01A* except M01AH*, M01AX25, M01AX99, M01AB55, M01AE52

A dose cut-toff can be defined, below this dose, the drug is not considered. For the two in-house NSAID this was: diclofenac: 75 mg, ibuprofen: 1200 mg

If on-demand prescription: number of documented intakes in the last 48 hrs: 6

Coxibes are considered as other risk factors for alert 11, thus not triggering an alert related to NSAIDs w/o PPI

**Considered prescription of anticoagulants**

ATC Codes: B01AA04, B01AA07, B01AE07, B01AF01 (dose must be >= 15 mg/day), B01AF02 (dose must be >= 5 mg/day, B01AB01 (dose must be > 10'000 E/day, to be checked manually as not assessable by automatically if continuous infusion), B01AB04 (dose must be > 10000 E/day), B01AE03, B01AX05 (dose must be >= 5 mg/day)

**Considered prescription of corticosteroids:**

ATC-Codes (Trigger Dose): H02AB01 (1 mg), H02AB02 (1.5 mg), H02AB04 (8 mg), H02AB06 (10 mg),

H02AB07 (10 mg), H02AB08 (8 mg), H02AB09 (40 mg), H02AB13 (15mg)

The corticosteroid prescription must not be stopped within the next 120 hours.

**Considered prescription of antiplatlets:**

ATC-Codes: B01AC06, B01AC09, B01AC11, B01AC13, B01AC16, B01AC17, B01AC21, B01AC25, B01AC27

**Platelet count:**

Presence of a laboratory value indication a thrombocytes count < 30 G/L within the last 10 days. In that case, restrictions concerning the minimum duration of concomitant drug prescription shall be ignored
